# Non-Invasive Inspections: A Review on Methods and Tools

**DOI:** 10.3390/s21248474

**Published:** 2021-12-19

**Authors:** Mubarak Alotaibi, Barmak Honarvar Shakibaei Asli, Muhammad Khan

**Affiliations:** Centre for Life-Cycle Engineering and Management, School of Aerospace, Transport and Manufacturing, Cranfield University, Cranfield MK43 0AL, UK; mubarak.m.alotaibi@cranfield.ac.uk

**Keywords:** non-invasive inspection, machine health, diagnostics, maintenance routines, radar sensors

## Abstract

Non-Invasive Inspection (NII) has become a fundamental tool in modern industrial maintenance strategies. Remote and online inspection features keep operators fully aware of the health of industrial assets whilst saving money, lives, production and the environment. This paper conducted crucial research to identify suitable sensing techniques for machine health diagnosis in an NII manner, mainly to detect machine shaft misalignment and gearbox tooth damage for different types of machines, even those installed in a hostile environment, using literature on several sensing tools and techniques. The researched tools are critically reviewed based on the published literature. However, in the absence of a formal definition of NII in the existing literature, we have categorised NII tools and methods into two distinct categories. Later, we describe the use of these tools as contact-based, such as vibration, alternative current (AC), voltage and flux analysis, and non-contact-based, such as laser, imaging, acoustic, thermographic and radar, under each category in detail. The unaddressed issues and challenges are discussed at the end of the paper. The conclusions suggest that one cannot single out an NII technique or method to perform health diagnostics for every machine efficiently. There are limitations with all of the reviewed tools and methods, but good results possible if the machine operational requirements and maintenance needs are considered. It has been noted that the sensors based on radar principles are particularly effective when monitoring assets, but further comprehensive research is required to explore the full potential of these sensors in the context of the NII of machine health. Hence it was identified that the radar sensing technique has excellent features, although it has not been comprehensively employed in machine health diagnosis.

## 1. Introduction

The application of maintenance routines is vital in any industry to ensure reliable asset operations. Maintenance aims to keep assets operating properly, save the asset itself by reducing failure times, minimise repair cost, ensure smooth production operation, and also to save lives and the environment. Most industrial disasters could have been avoided if proper maintenance had been done. For example, in 2010, the Deep-water Horizon (oil rig) caused one of the most massive oil spills in the Mexican Gulf and American history, eleven workers died and 3.2 million barrels of the oil spill from the well to covered more than 25,000 square miles of sea surface, leaving a lot of heavy oil and slug. The slug was around 10 million pounds from those states, and the impact on sea life was huge. The total loss for that disaster was 65 billion USD, all that could have been avoided if the main oil line killing valve had proper maintenance and condition monitoring systems [1]. The maintenance routine has three types of maintenance: breakdown or runs to fail maintenance, preventive maintenance and predictive and condition monitoring maintenance; their implementation has evolved significantly in the last few decades [2,3,4]. Besides the advantages afforded, breakdown maintenance has no requirement for prior resource planning or schedules. Limitations such as catastrophic damage to assets, costly repairs and replacements, lengthy stoppages, and delays in operations were the key factors prompting research into preventive maintenance. Preventive maintenance has helped to overcome the costly repairs and uncertainty associated with repair and replacement schedules. However, sudden failures, unnecessary repairs, and spare parts remaining useful life (RUL) are critical for reliable asset maintenance. Predictive and condition-based maintenance routines have been developed and implemented to avoid such failures and repairs. These routines involve many sensors and instruments, which generally require intrusive installation on the assets. To monitor the health conditions of machines, it has become standard practice to embed the necessary sensors and instrumentation during the assembly and commissioning of machines [5,6,7,8,9]. Furthermore, early fault diagnosis is the better plan with lower cost; researchers have classified the fault diagnosis process into three parts. The first part is fault detection, which calls the most basic task in the fault diagnosis mission, the second is fault isolation, which classifies the fault, and the third is fault identification, which determines the fault severity [10,11]. However, compromises are always needed to include these sensors in the design phase of the machinery. Besides new machines such as wind turbines, especially those offshore where each one-megawatt unit costs £3.3 million for installation, companies typically have very critical and expensive, old but effective operational assets [12]. These assets were originally designed with maintenance instructions based on corrective or preventive routines. Hence, embedding new sensing and instrumentation poses a challenge, but there is a clear need [13,14,15]. This need has prompted researchers to devise new techniques that can function without the need for any intrusive instrumentation and can work non-invasively. The research needs to be suitable to detect different types of machines, such as electric or fuel-driven; also, the location of the detected machine can provide more challenges depending on whether the detected machine is indoor or outdoor [16,17]. Therefore, this research will focus on finding a reliable technique that can be used on diverse machine types. Furthermore, this research seeks a technique that has an easy installation to detect critical locational objects, such as giant machines and wind turbines, and a higher accuracy, with a longer sensing range to measure and monitor gearbox damages and shaft misalignment during machine operation without interruption, and without using space in the machine. Hence, radar sensing techniques were proposed to fill the gap since nowadays radar has become part of our daily life; almost everyone on earth uses one of the radar applications every day, if not every single minute. The excellent features of the radar signal include a safe signal for human use, a long-range and fast signal that can travel, and the RF signal’s capability to resist weather difficulty and pass through many types of isolation. Radar technologies have been used widely, especially by the military where they have devoted radar technology to enhance their defense ability, and in airports and marines, which use radar in their daily activities. Hospital equipment uses radar in many non-invasive detecting devices. However, the industrial sector has the lowest advantage of radar technology. The radar operation principle for both radar types, pulse and continuous wavelet (CW), is based on transmitting electromagnetic waves with different wavelengths, amplitude, and frequencies to detect the target and send echo signals back to a separate receiving antenna as in CW radar types or to the same antenna like pulse radar. However, conventional radars cannot detect target movement, and so the Doppler feature assists the radar system to classify object movement.

The radar sensing technology has advantageous features to transmit and receive signals with high range and detecting quality. The methodologies of the project will determine the radar’s ability to detect machine health in a non-invasive manner, using radar sensor to detect machine displacement due to vibration caused by machine fault in shaft misalignment and gearbox teeth damage.

In this paper, tools and methods that can be used to perform machine inspections in a non-invasive manner are critically reviewed from the published literature. In the absence of a formal definition of NII in the existing literature [18,19,20,21,22,23,24,25,26,27,28,29,30,31,32,33,34,35], we have categorised non-destructive by non-invasive tools and methods in two distinct categories. Later, we describe the use of these tools and methods under each category in detail. The unaddressed issues and challenges are discussed at the end of the paper. The conclusions suggest that one cannot single out a NII technique or method to perform health diagnostics for every machine efficiently. There are limitations associated with all of the reviewed tools and methods but it is possible to select the best option if the machine operational requirements and maintenance needs are considered. It has been noted that sensors based on radar principles are highly effective for monitoring assets in remote locations or from a distance [36] but further research is required to explore the full potential of sensors in the context of NII machine health diagnostics [37,38,39,40,41,42,43,44,45,46,47,48,49,50].

## 2. Definition of NI Inspections (NII)

The existing literature on Non-Invasive Inspection (NII) tools and methods shows a difference of opinions among researchers to define NII. Some have categorized it as nothing but Non-Destructive Testing (NDT). In contrast, the definition of the NDT is the technique that tests and diagnoses the part characteristic without damaging the original part, which leads to identifying that the NDT can be an invasive tool but not destructive, where the others are strictly restricted to define NII with the features of non-contact-based sensor installation. However, it is apparent that any NII comes under the domain of NDT, but all NDT tools are not NII in their work operation. More precisely, we can categorize NII into two types, and their definitions are given below.

### 2.1. Type-1—Definition: Non-Contact-Base NI Inspections (NCNI)

**Figure 1:**
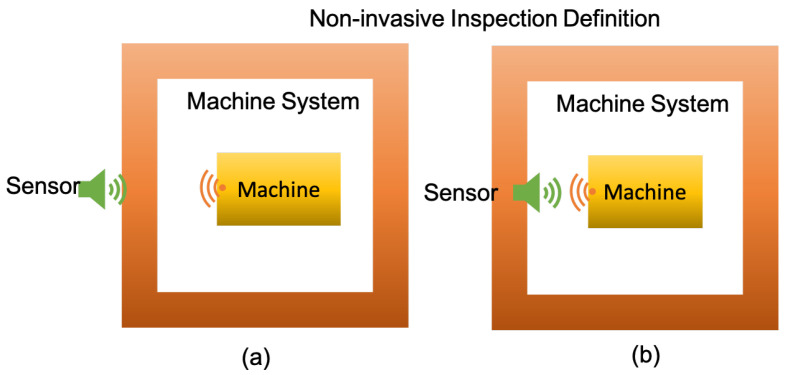
Non-Invasive definition (**a**) non-contact based and (**b**) contact based.

The sensing element does not require a physical contact with machine (i.e., under inspection) or within a machine system (i.e., structures and components have no contact with the machine nor the machine system under inspection) as shown in Figure 1a.

### 2.2. Type-2—Definition: Contact-Based NI Inspections (CNI)

The sensing element does not require physical contact with machine (i.e., under inspection) but it is installed within a machine system (i.e., structures and components directly in contact with the machine system under inspection) as shown in Figure 1b.

## 3. Tools and Methods of NI Inspections

The study aims to define NII tools to diagnose machine health based on sensing incorrect dynamic rotational frequencies and vibration due to the harm that occurs to static and rotating equipment in machine parts.

### 3.1. Non-Contact-Based Sensing

The non-contact based sensing, as it was defined earlier, is a type of sensor which does not require to be attached to the machine body nor to the machine system.

#### 3.1.1. Radio Frequency

**Figure 2:**
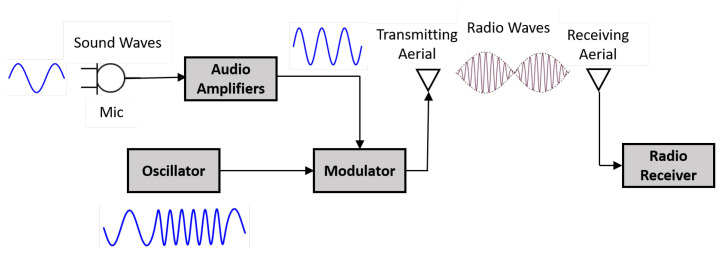
General principle of radio transmission [53].

**Figure 3:**
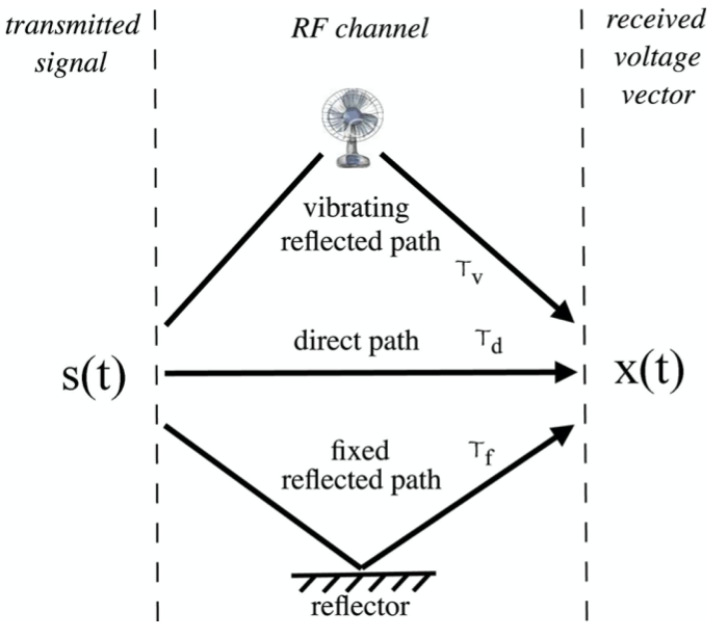
Polarization mode dispersion (PMD) caused by multi paths [51].

Radio Frequency (RF) implementation has significant usage in modern areas, like mobiles, car sensors, radio stations, GPS, and others. The RF inspection uses electromagnetic sine waves generated by a sensor and maintained by an oscillator to produce a high-frequency signal for longer distance transmission. The transmitted signal has different oscillating frequencies and amplitude emitted from a transmitting channel ($T_{X}$) antenna and detected by a receiving channel ($R_{X}$) antenna. The $T_{X}$ and $R_{X}$ working principle (Figure 2) encourages researchers to inspect a machine by observing the difference in emitted and received signals in a non-contact manner. For example, Mueller et al. used RF-based inspection to monitor the vibration of a desktop fan. They used the polarization mode dispersion (PMD) phenomenon caused by multipath in the propagating environment to separate the vibration signals of the fan as shown in Figure 3. They transmitted a signal at a frequency of 2.1 GHz that travelled in multipath, including the fan case or support, and was later received by the receiver antenna. The fan vibration signal was extracted by averaging the bandwidth of the PMD responses in both the time and frequency domains. In addition, the full bandwidth of PMD responses were analysed to describe a particular rotational orientation of the fan blade. The key limitation of this method is the transmission of the generated signals in non-metallic materials which can influence the reflected or dispersed signals available for the receiver and hence make the vibration measurement inaccurate [51,52] RF also measures and detects multi-machine simultaneously.

**Figure 4:**
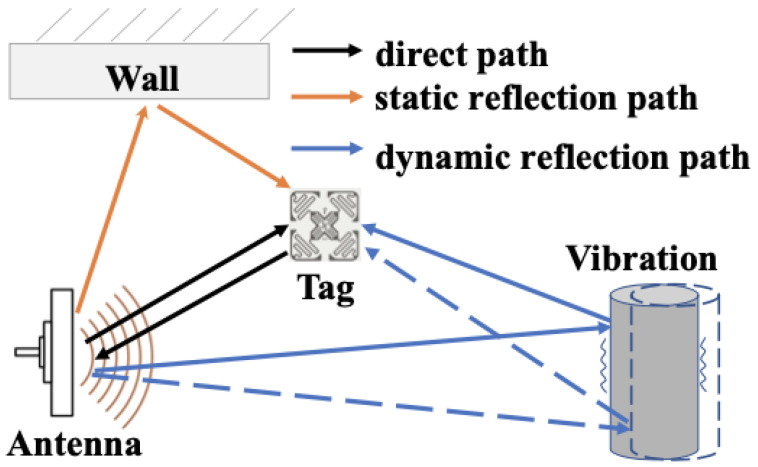
Vibration model [54].

**Figure 5:**
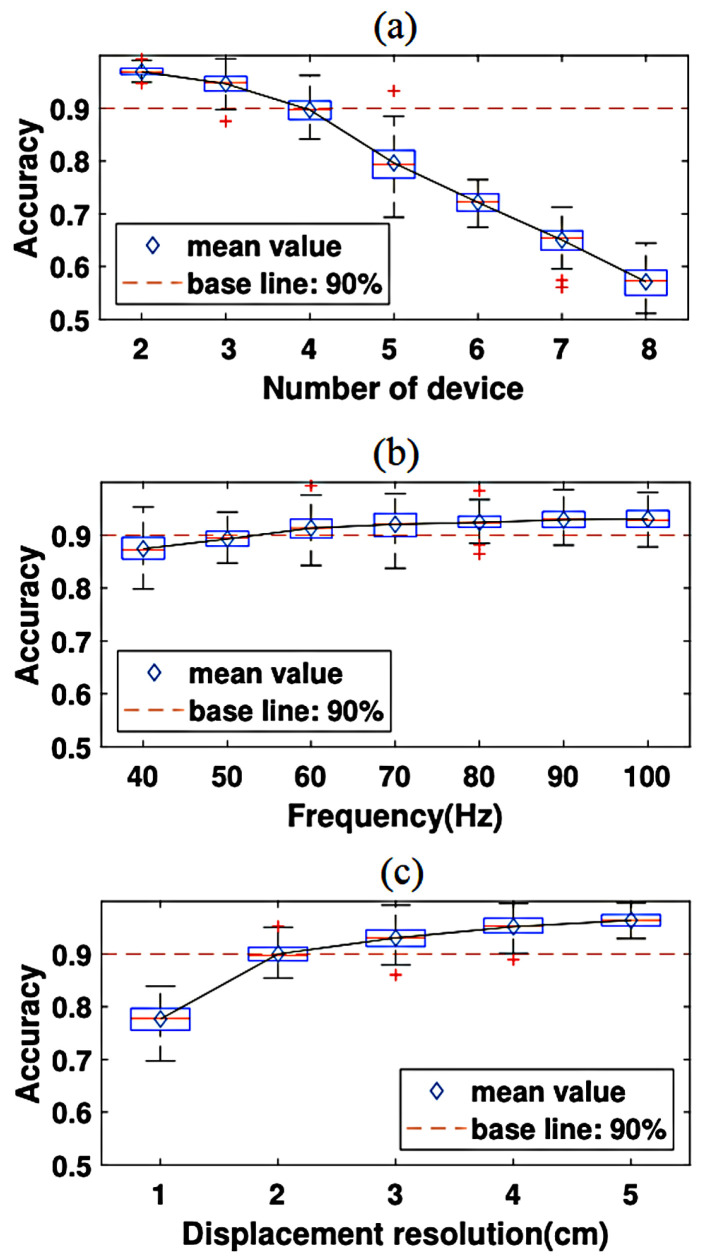
Identification results for: (**a**) number of devices, (**b**) frequency and (**c**) displacement resolution [54].

The existing RF-Ear technique was proposed to detect multi machines simultaneously working on different frequencies up to 400 Hz. A low-cost, radio vibration-sensing RF-Ear was applied using Impinj Speedway R420 and RFID Tags. The proposed technique was based on a three-path signals principle. Three signals were emitted from the transmitting antenna: one for the wall, the second for the tag with 100/100 reflection and the third for the object with vibration, as shown in (Figure 4). The implementation of the method faced several technical challenges, such as the sensors’ workability being limited to 40 Hz, an overlap in signals, and classifying the amplitude of machine vibration. The first challenges were overcome by introducing a Multi-Vibration Orthogonal Matching Pursuit (MVOMP) approach to recover the low-ranking signal by the method of voting and employing a scaling factor. The second challenge was to avoid the mixture of signals from multi-devices, which was achieved by using Gaussian noise. Gaussian noise was used to segment machine signals in the frequency domain while adding Additive white Gaussian noise (AWGN) increases the value of noise signals over the desired signals, and by removing AWGN signals, the value of the desired signal can be detected easier. For the third challenge, Short-Time Fourier Transform (STFF) was used to classify the amplitude of each machine’s vibration signal by building a spectrogram as a fingerprint. However, the identification scheme used for the devices was based on the crucial observation of long-term data collection to create a machine signal fingerprint. The fingerprint signal will be distinguished by STFT, with the window size set at 2 s and the overlap segment set at 0.2 s, and the result saved as a heat map image. The features of the fingerprint are extracted using Convolution Neural Network (CNN), which has an advantage over K Nearest Neighbour (KNN) and Support Vector Machine (SVM) in classifying the amplitude of each machine vibration. The work achieved the following results in identifying the number of machines: 90/100 were achieved, with seven out of eight devices being detected (see Figure 5a). To achieve 100/100, the number of devices would need to be a maximum of four. In depicting the impact frequency diversity, the accuracy slightly increased because of the better detection of the high central frequency (Figure 5b). For the displacement identification, the technique can detect a 2cm loosed screw with an accuracy 90/100. (see Figure 5c). The RF principle was defined above as the transmission of signals to a remote area, but if the $T_{X}$ and $R_{X}$ are in the same location, it is considered radar technology. The original use of radar was to detect objects such as ships and aeroplanes. However, the features of radar have inspired researchers to expand its usage [54].

#### 3.1.2. Radar

**Figure 6:**
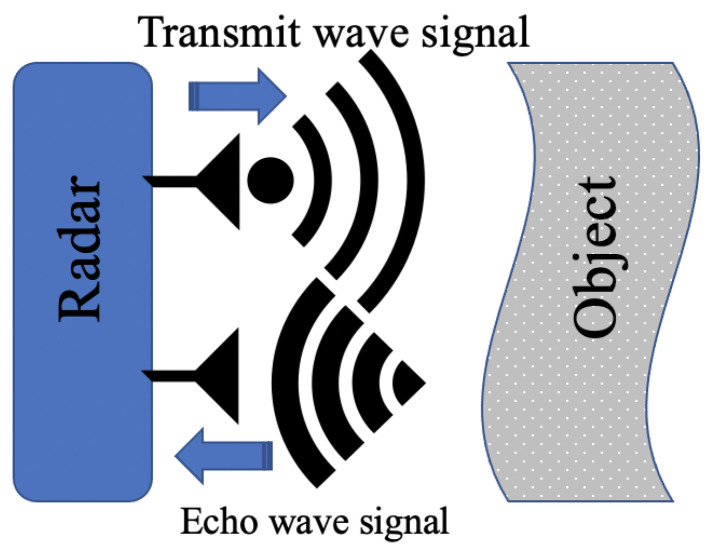
Operational principle of radar.

**Figure 7:**
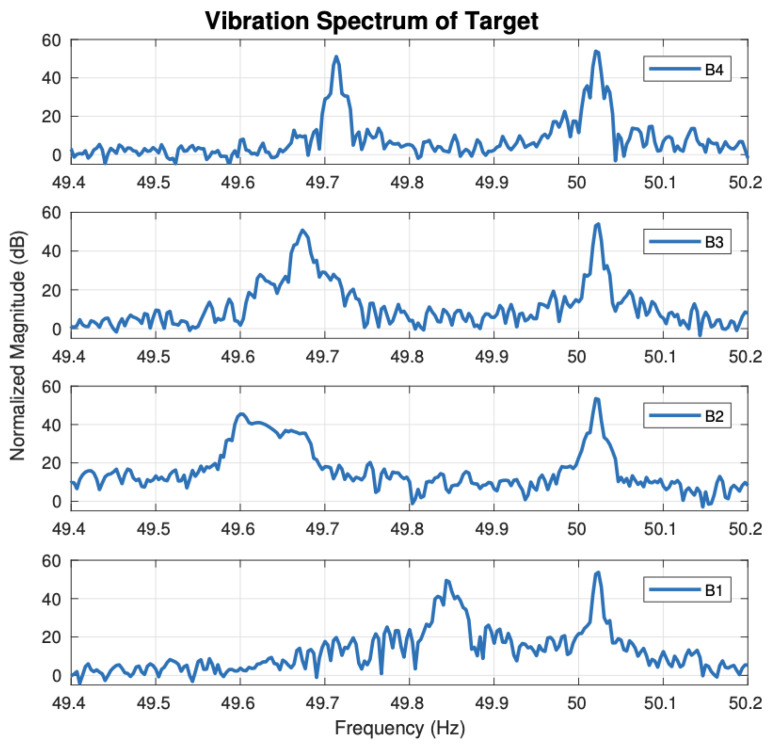
Vibration spectrum showing difference in multiple harmonics for each scattering matrix element [38].

Radar technology is an extension of radio frequency technology; it has the same operation principle but with some variations. RADAR is an acronym derived for Radio Detecting And Ranging, the basic principle of radar operation has not changed since it was devolved to detect marine ships in the early days, but radar design has undergone significant enhancement in general and in the received signal resolution due to development in digital technology and signal processing evolution. The radar is designed to detect object location, speed and size using the principle of the echo phenomenon, by generating and sending electromagnetic waves at light speed, and then detecting and analysing the returned signals as shown in Figure 6. The form of electromagnetic signals, wavelength and frequencies numbers are designed based on the type of information required from the objects, such as speed, size and distance, and radar has the ability to detect objects in the long distance. Lately, radar applications have gone beyond the conventional use as a marine and aeroplane detection instrument to be part of many industrial and daily live applications. These applications include use in hospitals for medical testing and scanning tools, cars sensing detectors, houses and industrial detection and monitoring security systems, and also as a part of industrial measuring sensing instruments. Radar has had significant implementation development during its history of use [45]. Researchers have used the features of radar to execute different tasks used Ultra-Wide-Band (UWB) radar to diagnose rotor machine bearing faults [50]. The method was based on projecting a high-frequency signal (10.5 GHz) to the object and analysing the reflected signal using data acquisition (SIGVIEW) software, with a sampling rate of 10 kHz. The acquired signal is sent to the Software Phase local Loop algorithm (SPLL) to obtain an error signal with Fast Fourier Transform (FFT), STFT, and Rotational Dilation Wavelet Transform (RDWT). The FFT analysis indicates the power spectra peak value but did not show the rotor harmonics, therefore, STFT was used to classify signal faults as shown in Figure 7, and RDWT was applied for bearing’s outer race and ball fault harmonics. The reflected signal energy increased from 4.72/100 to 5.82/100 with an increase in the number of errors. Later, in 2020, they published another academic paper in which a similar method was applied, but it was used to diagnose multiple machine faults. In that study, the reflected signal increased from 2.19/100 to 4.72/100 for the rotor bar and bearing in the no-fault condition. However, in the fault condition, the energy increased from 2.7/100 to 14.6/100 [48,49,50]. Radar features can be used to detect multi-machine faults simultaneously.

Radar techniques can detect and inspect the health of multiple machines simultaneously. Hershberger et al. used a dual-polarisation radar system NI-564R (VST) 250 MS/s. The reflected signal from machines is multiple and complex sinusoidal signals with different magnitudes and phases. Therefore, the analysis technique requires time-frequency characterisation to determine the number and frequency. Furthermore, a spectrum estimation technique was required to estimate the vibration spectrum for each component using Discrete Fourier Transform (DFT). The technique detected the four motors with a fundamental vibration frequency of 50 Hz and the bearings which have problems are three, Figure 7 shows that bearings (B1 and B3) were damaged, and bearing (B2 and B4) were less damaged [46]. The authors believe that the radar sensing detecting technique provides good inspection results for multiple machines simultaneously.

**Figure 8:**
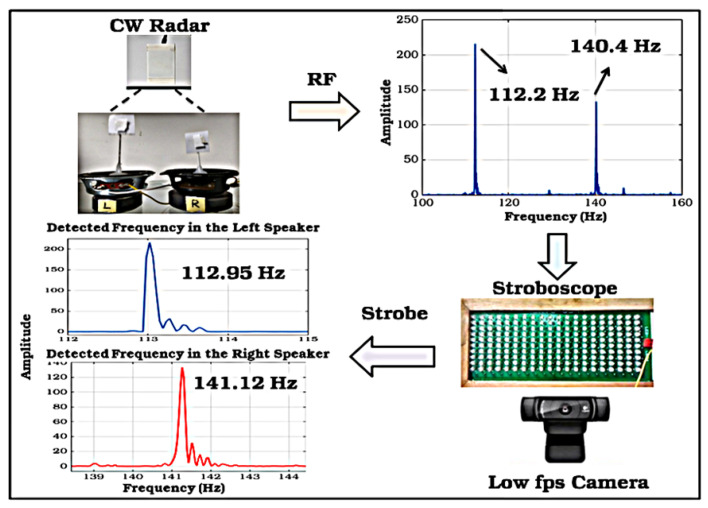
Result of vibration test using speakers [43].

A comparison has been made by researchers to demonstrate the efficiency of radar by comparing a CW HB-100 radar Doppler spectrum with a camera-image technique (CIT) associated with the optical-stroboscopic system to detect machine vibration and shaft misalignment, wobbling motion and to provide spatial information. The acquisition system used an MSP432 micro-controller to control radar trigging and stroboscopic lighting. The experiments were conducted in a dark area to avoid light noise. The work was executed in different phases: the first phase was to detect multiple linear motions by using two sound-speakers, with 10 microseconds detecting duration for both radar and camera. In the second phase, for rotational motion detection, an experiment using a water pump to detect single rotational motions and two motors for multiple rotational motions. In the third phase, an unbalanced, wobbling motion fault was generated in the measured motor. A study aimed to compare the radar sensing capacity with other types of sensing to evaluate radar efficiency. The results of the experiments showed that, for multiple linear detection of the two speakers supplied by 113 Hz and 141 Hz, the Doppler-spectrum detected 112.2 Hz and 140 Hz. The CIT detected 112.95 Hz and 141.12 Hz, with errors of 0.04/100 and 0.09/100 as shown in Figure 8. In the single rotational detection, the radar obtained 408.23 Hz (motor frequency 40.82 Hz × 10 blades), and the CIT observed 40.13 Hz, with an error of 0.33/100. The multiple rotational Doppler-spectrum provided two peaks, 93 and 246 Hz (3 and 4 blades), and the CIT process obtained 30.2 and 62.2 Hz, with errors of 0.32/100 and 0.67/100. The radar sensing technique has been compared with other well-known techniques to evaluate their efficiency, as shown in Section 4.3. It is clear that the radar sensing technique has provided reliable results [36]. The radar principle is based on emitting and retrieving signals, and the ultrasonic works similarly, but with the main difference in measuring distance range.

#### 3.1.3. Ultrasonic Sensing

**Figure 9:**
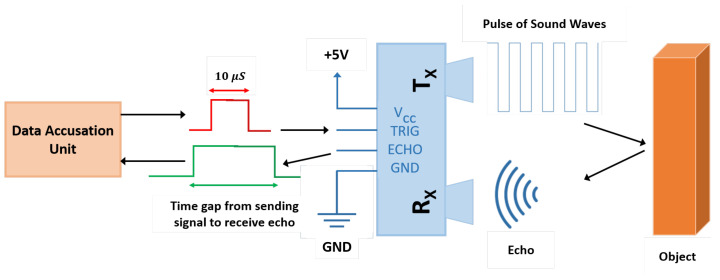
Ultrasonic principle [55].

**Figure 10:**
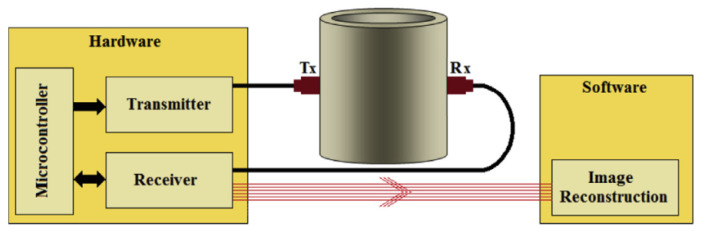
Block diagram of an ultrasonic tomography system [56].

Ultrasonic sensing works on the same principle as radar. It sends high-frequency pulses signals frequently at the airspeed to strike objects which are called trig signals. The echo signals are the reflected signals (Figure 9). By dividing the signal time by two, distance can be measured with an accuracy of 0.33/100 within the temperature range of 0 to 50 ${}^{\circ }$C [55]. Additionally, the industrial process used the ultrasonic sensing principle to detect gas bubbles in steel pipes by using ultrasonic tomography (UT). This technique requires hardware and equipment such as a micro controller, transmitting and receiving circuits with $T_{X}$ and $R_{X}$ sensing elements and software as shown in Figure 10. The technique is based on generating pulses with a resonance frequency from the micro controller and sending an electric signal to transmitter $T_{X}$, which is installed on the detected pipe. $T_{X}$ converts the electric signal to ultrasonic waves, $R_{X}$ in the other side of the pipe will receive ultrasonic waves then convert it back to an electric signal. However, during the travelling of the signals through the pipe sides, the signal is impacted by the liquid and gas media inside the measured pipe. The signals are then converted back to electric signals, passing through the receiving circuit to filter the noise, then constructing the image by using software such as the linear back-projection algorithm [56].

**Figure 11:**
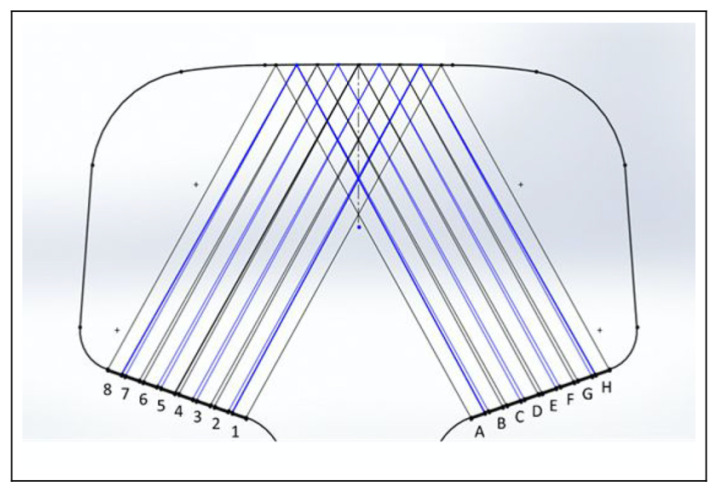
Pitch-catch ultrasonic measuring technique: The pulsers are labelled 1–8 from the inner bound to the outer bound, and the receivers are correspondingly labelled A to H. [57].

The conventional use for ultrasonic sensing was to detect metal thickness in a particular location, but guided wave ultrasonic testing (GWUT), which has a frequency range from 20 kHz to 15 MHz, can be implemented to inspect over longer distances. GWUT has various uses, such as diagnosing the thickness of oil pipes, storage tanks, rail tracks and wheels and rails. The existing ultrasonic techniques, such as ultrasonic guided waves (UGWs), were insufficient to detect the lifespan of wheels and railways during operational use. Therefore, ultrasonic sensing has been modified to investigate in-service rail structures. The pitch-catch ultrasonic detecting technique has been proposed. This technique uses two sets of sensors, each with eight 10 MHz piezoelectric sensors arranged linearly, as shown in Figure 11, and bonded onto the rail surface. The sensors on one side of the rail emit pulses and receive the reflected signal from the top surface of the rail on the other side. The transmitter sensors are numbered 1–8, and the receiver sensors are marked A–H. Theoretically, each emitted signal can be received by all sensors, but by using the ray-tracing software, the receiving method is optimized to H1, G2, F3 etc., instead of H123, ..., G123, etc. The technique was carried out using a full-scale dynamic test at a speed of 5 mm/s, the method provided important information about the interface of the wheel and the rail at low speed (5 to 20 mm/s), but its use at higher speed still needs system enhancement. Despite this, ultrasonic sensing has been used in different areas such as security systems, handicap help and building foundation inspection [57,58].

#### 3.1.4. Camera Based Imaging

**Figure 12:**
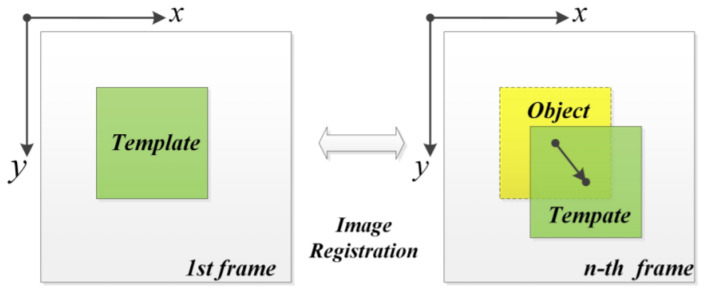
Process of implementation [59].

**Figure 13:**
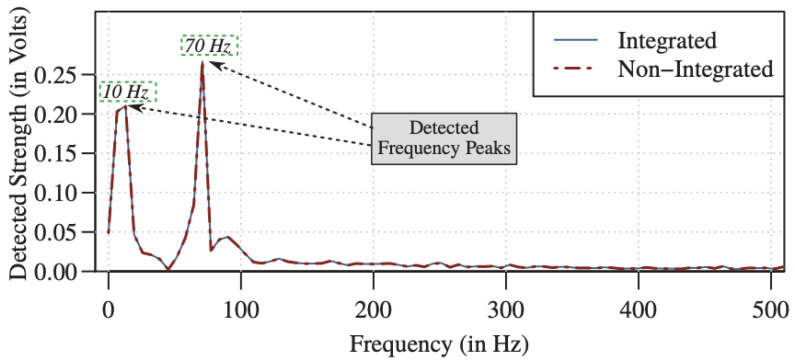
Result of combined communication signal and sensing [60].

**Figure 14:**
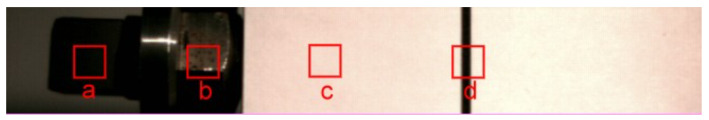
Image with and without white sheet paper. The squares a and b enclose different parts of the accelerometer and the squares c and d contain locations of the white paper with and without the black straight line. [61].

The imaging camera principle is based on a phase-based optical flow for image processing to measure the real-time motion of objects. This is established by capturing video to measure an object and ensure the moving target is in photographic range. Then, the template image is cut in the first frame, and the object images are cut to compare the resulting video image sequence at the same position. Finally, the cross-correlation matrices are calculated using the 2D Discrete Fourier Transform (DFT) of every object and template image. The maximum value is then recorded (see Figure 12) [59]. A modified Taylor approximation refinement and localization refinement is used for efficient sub-pixel refinement. The technique is employed to detect and monitor industrial machines. The technique adopts a strobing light with a low frame rate camera to measure high-frequency vibration. Visual light communication concepts based on a strobing light and a low First Person Shot (FPS) camera with a data rate of 80 kB/s have been used to measure object vibration using narrow depth optical pulse and adjustable frequency. The optical sample signal modulates the object vibration, then shifts the component using the Nyquist frequency and applies frame-to-frame comparison to illustrate the object. At the same time, the signal is transferred through a wireless communication system using a radio-based GNU (radio software). The technique lines up the strobing light with the communication system to activate both at the same time. The proposed technique aimed to investigate the influence of combining strobing light with a camera and wireless communication to measure the signal. It demonstrated a small error between the strobing system, i.e., integrated and non-integrated strobing pulse, which detected in the testing of 10 Hz and 70 Hz signal (see Figure 13) [60]. Jeng and Wu, 2012, applied a similar method using the Prosilica CV640C CMOS high-speed camera with a green LED, but added a white paper sheet with a black line on the vibration system surface. The white sheet enhanced the imaging signal as shown in Figure 14. The technique was used twice to detect slight helicopter vibration using a high-speed camera and a microphone, and a second objective was to obtain the arterial pressure signal from a young man’s neck. The technique detected the helicopter vibration but not the man’s arterial pressure [61].

**Figure 15:**
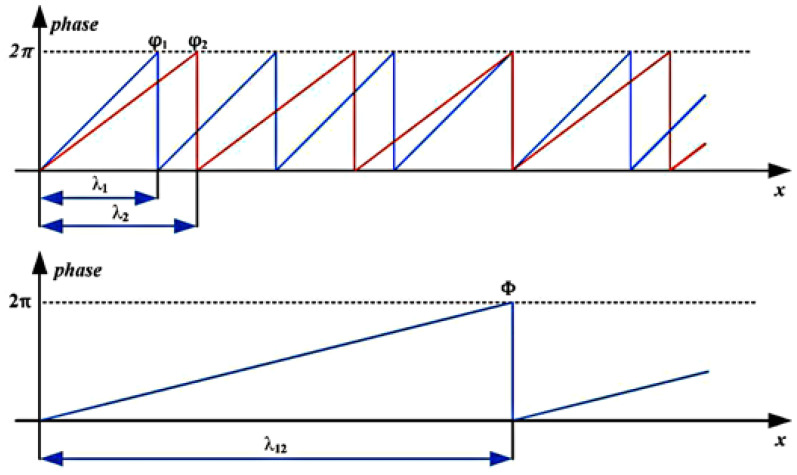
Heterodyne principle. $\phi_{1}$ and $\phi_{2}$ (phase 1 and 2) are the wrapped phase functions and $\lambda_{1}$, $\lambda_{2}$ correspond to the unwrapped phase function [62].

Han et al. [62] introduced multi-frequency phase-shifting-based (PSP) 3D sensors for the measurement of environmental vibration error. The principle of the technique is based on a stereo vision by projecting fringe patterns onto the measured body for scanning. Phase constraint and epipolar constraint were used to locate the corresponding points in two cameras, and for the phase-shifting image sequence, a warped phase map was also applied, with captured images recovered from the Gray values of the same pixel in different frames. The PSP works by projecting sinusoidal fringes horizontally with the projector image frame in constant phase-shift, in which the cameras capture phased-encoded images. However, the sequence of images may be affected by environmental vibration and can be analysed by understanding the sequence’s structure. Therefore, employing a combination of multiple frequencies to produce frequencies lower than any of those by applying the multi-frequency heterodyne to adjust cycles until only one cycle achieves in the entire field of view (see Figure 15). Identifying the magnitude of the additional phase shift determines the strength of the streak, which means the phase shift magnitude depicts the vibration magnitude, determined by extracting the region of interest (ROI) by applying a FT map [63,64].

#### 3.1.5. Acoustic Emission Sensing

**Figure 16:**
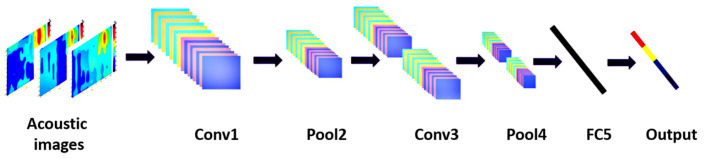
Transfer the acoustic signal to image (CNN model) [69].

Acoustic emission (AE) is implemented to detect sounds but, in the diagnosis of machine health, it is used to detect and analyse specific signals of machine noise to compare and evaluate for fault diagnosis. Generally, noise is a part of machine operation, but affected machines emit a higher level of noise. Therefore, the principle of AE is used to capture the emission phenomenon wave by microphone, then analyse the ROI. Malfunctions of motors generate different types of noise based on the location of the fault. However, a combination of noises can be mixed in one signal. The electromagnetic source of noise is based on stator and rotor, and the mechanical source of noise comes from shafts and bearings rubbing, imbalance status, and aerodynamic noise from a cooling fan. To demonstrate the AE coefficient in detecting machine faults, it is necessary to evaluate the efficiency of acoustic technique with other sensing techniques. Experiments have been done by researchers to compare a vibration accelerometer with a sensitivity of 500 mV/g, a 50 mV/Pa microphone and an AC sensor with a sensitivity of 0.1 A/V in detecting a tooth fault in a gearbox. Signal processes analysis (SPA) techniques were used, such as stator Power Spectrum Density (PSD), a high-resolution acquisition system based on the Welch technique and envelope analysis, to analyse vibration and acoustic signal. The fault was in the pinion tooth surface which would generate mechanical shock pulses. The fault was sensed by vibration and acoustics. However, the current analysis methods used did not detect the fault. The SPA involvement enhanced the AE in diagnosing the machine faults [65]. Various SPA methods have been used with AE, to detect problems during variable loads and non-stationary techniques such as the multi-stages technique. The technique scatters the signal and filters it, then uses the scattering coefficient for a better classification rate, after which it reduces the dimensions of the feature space by linear discrimination analysis (LDA), and diagnoses the fault by support vector machine (SVM) with radial bases function (RBF), and the method detects 98/100 [66]. For the same context, a combination of SPA based on spectral analysis, such as Intrinsic Mode Function (IMF) with a Fast Fourier Transform algorithm (FFT), was used to detect specific faults in a rotating machine’s bearing and unbalanced parts by use of a microphone JST model CX-509 type and a signal amplifier and digital converter. The combination of SPA technique detected bearing faults and unbalancing based on the power-spectral-density of the machines sound signal, however, the noise still had a high impact [67]. It is likely that a machine fault has a vibration impact and other effects on other parts, for example, a bearing fault has impacts on the shaft’s vibration sensing. Therefore, to detect multi-faults in machines, the Probabilistic Neural Network (PNN) is used to classify the signals, with lab-View software used to control data acquisition. The method uses accelerometer type PCB Piezotronics and ICP sensors to achieve higher accuracy and minimisation of error values [68]. Moreover, to enhance acquired signal analysis, AE can adopt the Convolution Neural Network (CNN), Stochastic Line Search (SLS) and constant-Q transform (CQT) to utilise the time–frequency-domain representation of the non-stationary signals and transfer the acoustic signals to images using the Wave Superposition Methods (WSM) (see Figure 16) [69]. However, noise has the highest impact on the acquired signal.

**Figure 17:**
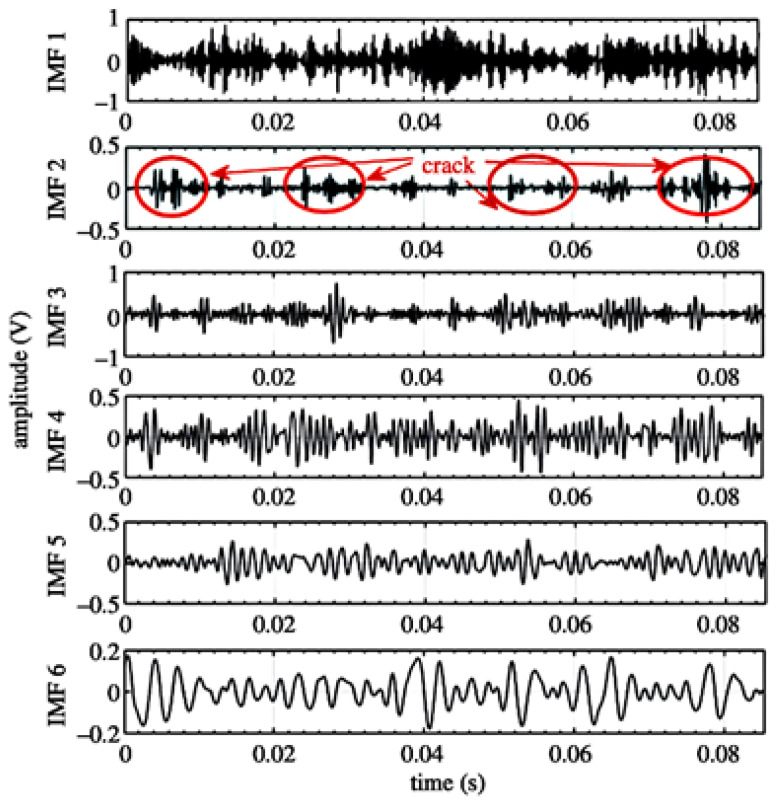
Fault detection using CEEMDAN method [70].

Several SPA techniques can be used to eliminate the influence of noise, the Ensemble Empirical Mode Decomposition (EEMD) has an advantage signal process analyses for fault detection. However, it still has some weakness due to the residual noise, and techniques such as Complete Ensemble Empirical Mode Decomposition (CEEMDAN), Intrinsic Mode Function (IMF) and white Gaussian noise can enhance noise elimination. These techniques showed an ability to detect the fault in the gear, as shown in Figure 17 [70]. To improve the accuracy of AE sensing, a redundant AE sensor needs to be installed, however, this will increase the acquired data size. Conventionally, to reduce the acquired data size, a joint application based in Blind Source Separation (BSS) to separate mixed sounds, and Compressed Sensing (CS) to reduce the amount of transmitted data, were used. However, applying a Compressible Source Separation (CSS) scheme shows higher quality of separating the acquired data [71].

**Figure 18:**
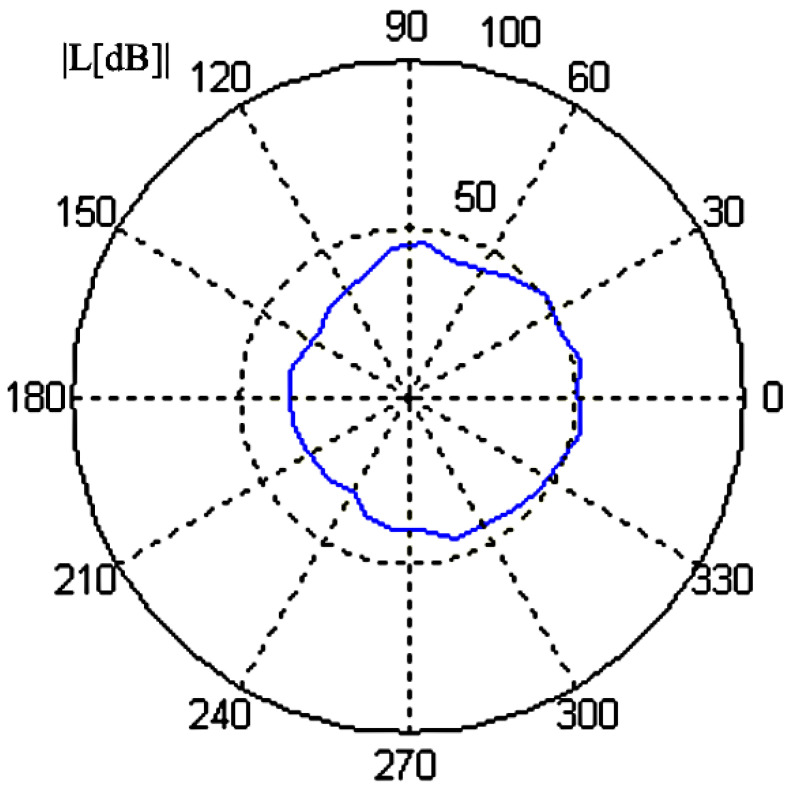
High level of noise position at 120 degrees [76].

Another SPA technique is used for segregating data to minimise the data load capacity and divide the data into two parts, online and offline phases. The offline phase is related to the establishment of a CNN, whilst the online phase uses the CNN. The proposed technique is to reduce the input size for the CNN by reducing the number of FLOPs and the inference time by trimming needless components and using up-to-date CNN architecture. The method can detect 99.58/100 of bearing faults [72,73]. The AE technique can be applied to more than simply detecting a fault in a machine; it can also detect other equipment, such as the power switch of power transformers. The method has also been used to detect On Load Tap Change and amplified the received recording signal. The signal is analysed through extraction of Power Spectrum Density (PSD) using the Welch method (Welch PSD), and the detail of the energy coefficient is determined by using a Haar wavelet [74,75]. Furthermore, AE can be used to estimate cutting depth during the end-milling process by measuring the acoustic emissions generated during the drilling operation. Janda M, Vitek O and Skalka M, 2010, found that the high levels of noise are placed at 120 degrees from each other in the revolution graph chart as shown in Figure 18 [76].

#### 3.1.6. Thermographic Sensing

**Figure 19:**
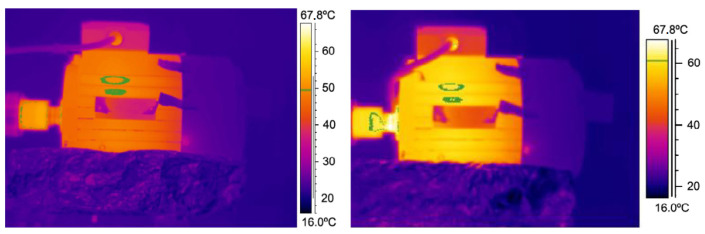
Comparison of a healthy and a non-healthy machine [79].

The infrared (IR) thermography sensing technique is a non-contact tool that measures the surface temperature of objects. The IR working principle is based on measuring emitted infrared wavelength from a detected object then transferring that signal into images, with the colour palette depicting each temperature. The technique uses several sensors, such as thermal imaging cameras, infrared thermometers and ratio pyrometers. The operation of a thermographic camera is based on creating images using infrared radiation. Machines may suffer from multiple faults simultaneously. Some techniques have difficulty classifying faults, but thermographic imaging can detect multiple faults in a machine. The first law of thermodynamics can be applied using an infrared thermographic, high sensitivity, long-wave thermo-camera FLIR S65 linked to a PC and operated by Therma-CAM to capture images that display a scale of colours representing the temperature in degrees (See Figure 19). Gini Coefficient (GC), Standard Deviation (SD) and moment of light are used for bearing fault analysis, and an infrared image based on two measuring lines is applied to enhance the classification of faults. The practice has two branches; the first branch focuses on the shaft alignment, and the second branch to classify the bearing faults. However, the limitation of that work was that the fault classification remains unsatisfactory [77,78,79]. Furthermore, the images feature can detect and locate faults where the colour code specifies the severity of the fault.

**Figure 20:**
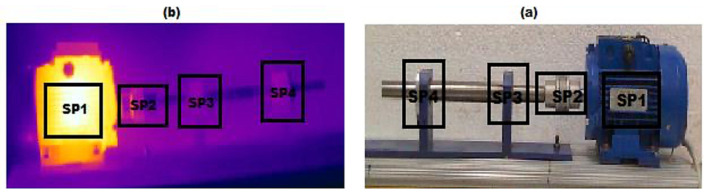
Motor and bearing thermogram: (**a**) experimental setup and kinematic chain and (**b**) thermographic image [80].

The colour code feature adds to fault detection and fault severity. The infrared thermographic technique can be used to detect the severity of the shaft’s misalignment using a FLIR 440 thermal imager. The two-dimensional signals method has the advantage of analysing the hot spot and detecting a small zone through its segmentation. The working principle is based on the thermographic camera capturing a digital image, with each pixel represents a temperature value as shown in Figure 20, then transmitting it to a PC equipped with MATLAB software for fault analysis and a Speeded Up Robust Feature algorithm (SURF) to determine the Regin Of Interest (ROI) of the bearing and its misalignment [80,81,82]. In the same context, rainbow encoded software can be used to convert the electric signal to the colour image when testing an air compressor using a FLIR 0.01C resolution camera fixed 80 cm above the object. The results approached 0.907 accuracy in detecting the three faults. However, the signal loses critical information during transfer; hence, effective communication is the main issue in detecting faults. An MLX-90621 thermographic sensor is used with a microcontroller kit to support Wi-Fi mode-bus and Xamarin software (compatible with IOS, Android and UWP). Communication model HC-06 is used to communicate between the MCU, the Bluetooth device, and the mobile to enhance the quality of the transmitted signal [83,84,85,86].

#### 3.1.7. Laser

**Figure 21:**
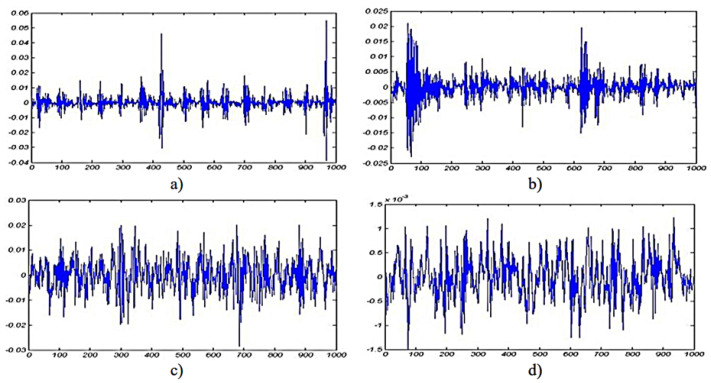
Different bearing faults: (**a**) inner race, (**b**) outer race, (**c**) ball fault and (**d**) healthy bearing [87].

Laser is an acronym for light amplification by the stimulated emission of radiation. The laser machine is a device that stimulates molecules or atoms to emit light; the light emits a particular wavelength and amplifies that light in order to produce a very narrow radiation beam. The laser was an outgrowth in 1916, then in 1928, German physicists observed the first simulation emission. There are different types of lasers, such as glass lasers, crystal, liquid, gases and semiconductors. The laser has been used in many applications, beginning in the early 1970s such as a light projector with different colours. The laser widely spread to be involved in daily life such as information transmitting and processing like supermarket scanners, optical sensors and fibre-optic communication systems. Furthermore, medicine has gained colossal benefits from laser technology, particularly in surgeries and tissue removal; also in eye treatment, stopping bleeding and many more usages. The military used high-energy laser technology to produce a high destructive power to destroy targets at the speed of light. Industry has benefited from laser technology as well, in applications such as surveying, and measuring the distance from the earth to the moon. Artificial intelligence technology is also used to determine and classify bearing faults in rotating machines with the help of the neural network (NN), by distinguishing the retrieved signal using discrete wavelet coefficient analysis. Furthermore, designating the use of standard deviation to identify fault type, the feature vectors design network uses the system input to characterise the fault located in four points: inner bearing, outer bearing, ball-bearing defective, and healthy bearing. This method achieves 0.99 performance for the inner and the outer bearing, as shown in Figure 21 [87,88]. Another costless laser application is the computer mouse, an optimising computer’s 2D optical laser mouse 800 dpi with dedicated software (a 635-nm 5-mW diode laser replaced the original one in the intense mouse beam) used to detect bearing faults. By projecting to the X and Y axis and using the time domain to position object vibration and frequency domain to identify components, Fourier analysis and Peak value can be used for complimentary analysis. The operation of the method relies on root mean square (RMS) and FFT algorithm values. Laser vibrometer has high accuracy, and by comparing an accelerometer to laser vibrometer in bearing fault and rolling element, the laser approach gives a good result similar to the accelerometer [89,90,91]. The laser also uses machine fault diagnosis to bridge the state diagnosis by determining bridge cables by sensing the cable vibration, as it has an extended measuring range from 30–100 m. Moreover, it has the flexibility to position itself due to its high accuracy.

### 3.2. Contact-Based Sensors

The NII contact based detecting tools as defined earlier are that the sensing devices which have to be attached to the measured machine’s body or to the machine system.

#### 3.2.1. Magnetic Flux and Voltage Sensing

**Figure 22:**
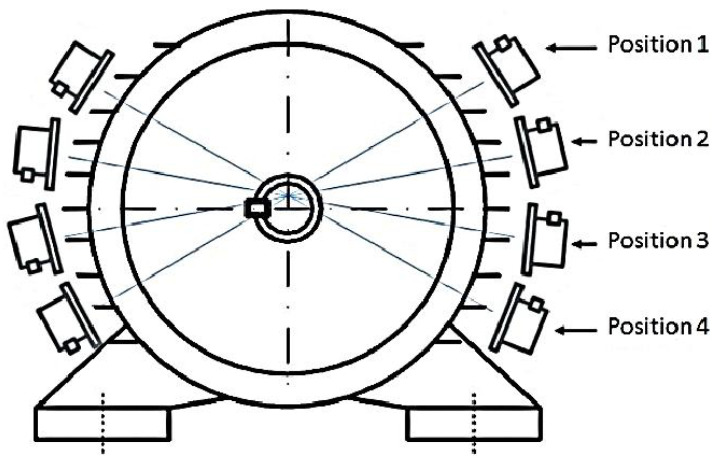
Four pairs of sensors [94].

**Figure 23:**
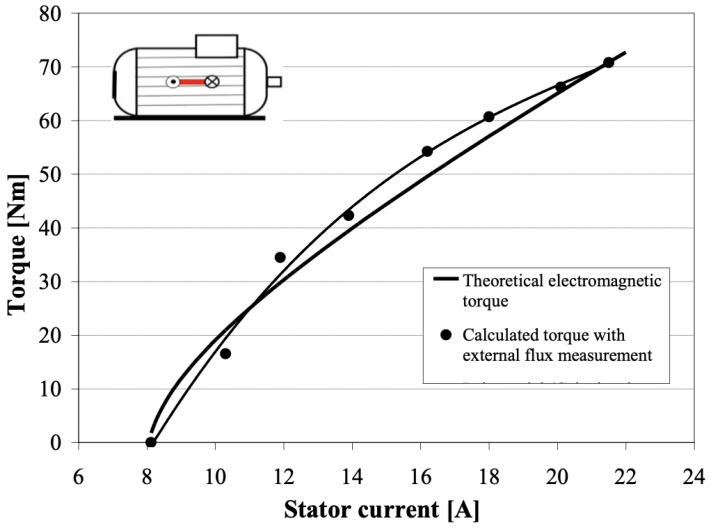
Theoretical and calculated torque [92].

Flux radiation or voltage sensing is a non-invasive contact-based technique. The flux technique is widely used in diagnosing faults, consumption and efficiency in induction motors. The method has a non-invasive feature as it is a non-interrupting technique, but it is also a contact-based technique. The principle of the technique is to measure a motor’s electromagnetic field (EMF) radiation, which is influenced by a motor’s loading and fault state. However, the location of the sensors also impacts the accuracy of the measurements. Early practices used two sensors placed at 180 degrees from each other, to diagnose inter-turn short circuit faults in flux coil devices by sensing generated EMF from the motor. The output of the two sensors was then compared. If both outputs had the same harmonic amplitude, then there was no fault, but if not, there was a fault, which could be detected by using a single-phase system, as shown in Figure 22. Moreover, to improve the measurement of the flux air gap, six sensors were mounded around a rewound induction motor. Later, the number of sensors was increased to cover most of the air gap around motors. Consequently, four twin flux sensors sat around the rewound induction motor. However, another technique placed the sensors against the motor yoke, using the tangential component to avoid flux air gap and end-windings. The TANG method helps to calculate the motor torque and the result are compared with the theoretical data, as shown in Figure 23 [92,93,94]. Diagnosing a single machine is a common practice, but manufacturers are interested in the testing of many machines simultaneously. Therefore, Giant Magneto Resistive (GMR) sensors were provided to detect faults in different machines simultaneously by using a very high sensitivity sensor and resolution to measure flux leakage. However, the enhancement in the signal process analysis added a significant evolution into the ability to the fault diagnosis process [95].

**Figure 24:**
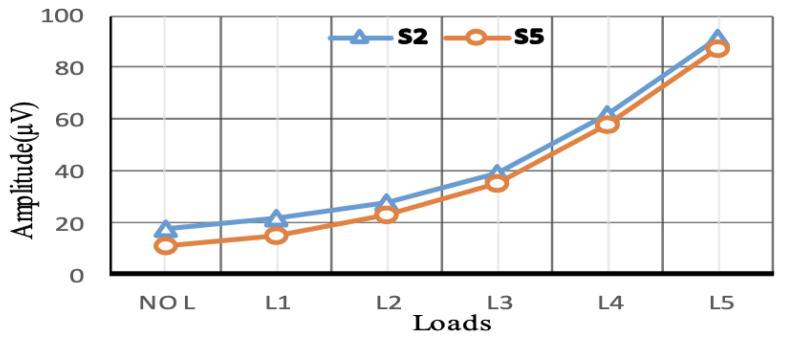
No fault detected [96].

**Figure 25:**
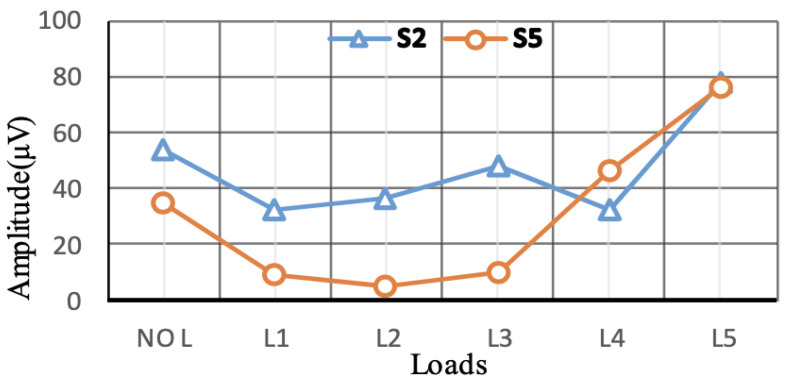
Fault detected [96].

Signal process analysis uses different techniques to analyse the acquired signals. For example, a motor’s spectrum magnetic field can evaluate the motor’s health without knowing the motor’s initial health state, by using load variation to perform fault diagnosis. The technique is based on applying the Belief Function Framework (BFF) to merge and represent the short circuit information (information representation, combine evidence and decision making). Furthermore, the provided data can be compared with a machine state identified as not defective (no-load and load case) when sensors provide harmonic amplitude in the same direction that show no fault but load change, the method achieved fault detection of 0.872 to 0.949, as shown in Figure 24 and Figure 25 [96]. Another SPA technique uses a finite element based on investigating the effect of the stator winding fault in motors. The inter-turn faults are also detected through the spike element of the components based on the Bedrossian theorem [97,98]. Furthermore, in order to diagnose machine faults during start-up and transient time, the Fast Fourier Transform (FFT) technique can be added to SPA implementation. The method uses EMF signals which are transmitted to a PC. The FFT is employed for stationary analysis, Short Time Fourier Transform (STFT) is used for start-up, and the Discrete Wavelet Transform (DWT) is used for spectrum analysis [99]. DWT is a useful and commonly used method, and it is also used in the current signal analysis (MCSA) technique as well. Additionally, for bearing faults in wind turbines (PMSG), SPA was investigated by estimating the speed of the turbine (at low rate) based on the angle of the voltage vector to re sample the vibration signal and the estimated rotor position by measuring the output voltage using a phase-locked loop (PLL). The voltage vector angler calculates the varying speed and Fast Fourier Transform to characterise the faulty components and detect outer race faults in bearings at different speeds [100].

#### 3.2.2. Machine Current Analysis

Motor Current Signature Analysis (MCSA) is a contact-based technique based on momentary changes in a motor’s current consumption. MCSA is widely used, even though it has a weakness in detecting multiple faults, especially during load change and different bearing faults, as stated earlier when discussing AE. Where AE and accelerometer sensors were able to detect bearing faults but MCSA could not [65]. An MCSA and thermographic techniques experiment was instigated using a FLIR S65 series infrared analysis camera and Thermacam Researcher software, and an MCSA sensor was linked to a Yokogawa DL-850 Scopecorder. The Scopecorder instrument transfers the signal to a PC fitted with MATLAB software to detect stator current during the start-up and steady states. This twofold method can measure the stator’s current signal at a sampling rate of 5 kHz and uses the registered time to analyse the MCSA frequency resolution [77]. However, the MCSA failed to detect bearing faults under the same conditions as infrared technology can detect faults, but employing SPA enhanced the MCSA technique in the detection of bearing faults in a machine. Of machine failures caused by bearing and bearing faults, 0.70 are characterised based on the fault location, such as inner race fault, outer race fault, and ball race fault. Therefore, each type of fault has a different detection method. Hence, a vibration analysis technique and MCSA were combined to detect Rolling Element Bearing (REB) faults. The technique used in [101] is based on the Equations (1)–(3) for frequency calculations in terms of the outer and ball bearing faults to analyse the time domain acquired signal.

(1) $$F_{i}=\frac{N_{b}F_{r}}{2}\left(1+\frac{d_{b}}{d_{c}}\cos\beta \right),$$

(2) $$F_{o}=\frac{N_{b}F_{r}}{2}\left(1-\frac{d_{b}}{d_{c}}\cos\beta \right),$$

(3) $$F_{b}=\frac{d_{c}}{d_{b}}F_{r}\left[1-{\left(\frac{d_{b}}{d_{c}}\cos\beta \right)}^{2}\right],$$

where $F_{i}$ is the inner race fault frequency, $F_{r}$ is the rotor frequency, $N_{b}$ is number of balls, $d_{b}$ is the diameter of ball, $d_{c}$ is pitch circle diameter, β is contact angle of ball, $F_{o}$ is the outer race fault frequency and $F_{b}$ is the ball fault frequency.

Fourier Transform is used to decompose the signals to the frequencies and spectral analysis with an Artificial Neural Network (ANN) to make output decisions. The method detected 0.98 of faults, but the vibration analysis method in that experiment approached only 0.85. However, MCSA still has difficulty analysing the machine’s states during the operation transit from zero speed to high speed and load variations accelerating from a steady state to full load. Hence, the Advanced Transient version (ATCSA) was used to detect faults during transit conditions MCSA. The method used in [102] applied Equation (4) for steady-state speed fault detection and implemented Equation (5) to detect faults at variable speed.

(4) $$f_{bb_{1}}=\left(1\pm 2k_{1}s\right)f_{s},$$

(5) $$f_{bb_{2}}=\left[\frac{k_{2}}{p}\left(1-s\right)\pm s\right]f_{s},$$

where $f_{bb_{1}}$ and $f_{bb_{2}}$ are two broken bars frequencies. *s* is the slip, $f_{s}$ is the supply frequency, *p* the number of pole pairs, $k_{1}$ is any natural number and $k_{2}$ is a positive integer (p,3p,5p,⋯).

**Figure 26:**
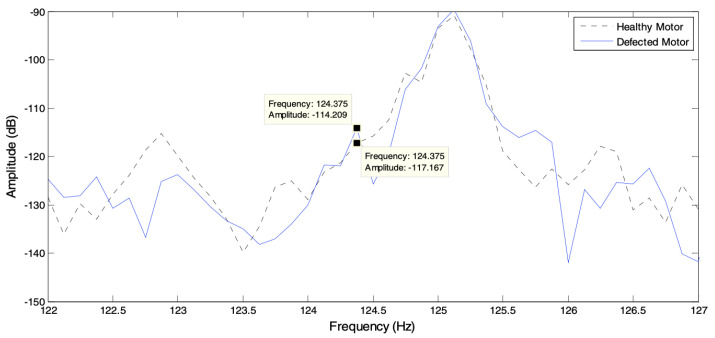
Localize analysis equation [104].

**Figure 27:**
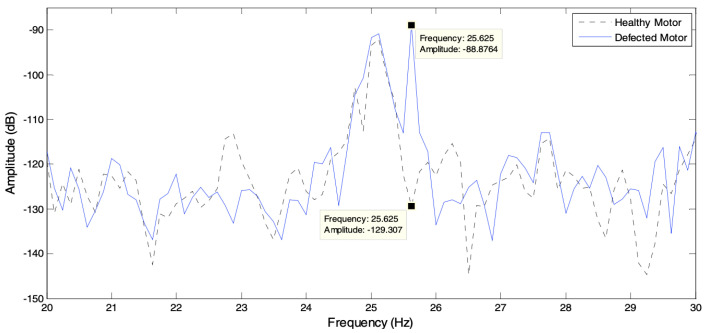
Park analysis equation [104].

Moreover, MCSA can depict problems in gear tooth surfaces and broken bars by FFT and SFFT based on steady-state analysis, but methods still have a drawback in displaying all frequencies. Hence, to overcome all those problems, Discrete Wavelet Transform (DWT) was used for broken bar detection and used a K nearest neighbor KNN algorithm for decision and classification [103]. The failure of the bearing was classified into two main types: localised and distributed faults. The localised analysis equation was applied to detect a distributed bearing fault, but the fault was not sensed (see Figure 26). Therefore, to observe the change in the signal’s amplitude related to the bearing fault, the technique treated the fault as a distribution fault and applied the Park vector analysis (PVA) Equation. The method operated by shifting the three-phase voltage and current then calculating the current and voltage modules, as shown in Figure 27 [104,105].

#### 3.2.3. Vibration Technique

**Figure 28:**
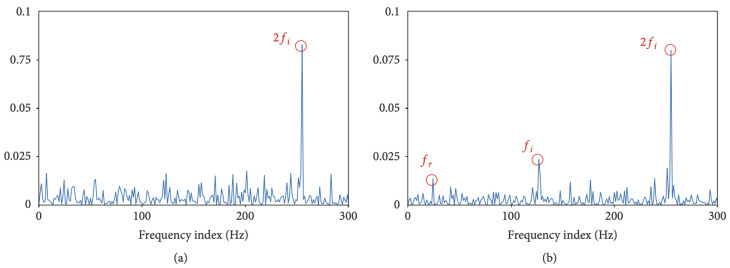
(**a**) Denoised signal by standard LP denoising method, (**b**) Frequency domain plot of denoised signal by AHLP denoising method [111].

The vibration sensing technique (VST) is one of the most commonly used techniques in machine fault diagnosis. It provides reliable and robust evidence of a machine’s condition with less noise impact. The principle of VST is based on measuring the displacement of the moving parts and the stationary parts. It uses piezoelectric sensor technology, which comes in different types, such as accelerometers, strain gauges, and velocity and gyroscope sensors. The vibration technique indicates current machine health, which helps to predict machine performance and efficiency. A practical measured pump’s flow rate was measured to indicate the pump’s performance using accelerometer mounted pad sensors, which were glued onto eight pump bodies using a wavelet fuzzy clustering programme. The experiment achieved 0.979 of determining the pump’s flow rate based on the condition of being lower or higher than 0.90 [106]. A conventional single-axis accelerometer for vibration measuring is time-consuming. Therefore, the visualisation of machine vibration motion is proposed. The proposed method simplifies the operating deflection shape (ODS) analysis to measure four points, two bearings on the drive side and two on the driven side. Using two accelerometers through the relative phase provides a phase between two measuring points for balance and misalignment analysis [107]. Furthermore, vibration sensing detects shaft misalignment and bearing wear with higher accuracy. A low-cost vibration sensor Micro Electro Machine System (MEMS) accelerometer was used to achieve a good result in detecting shaft misalignment at high speed, but at low speed the characteristic was not well identified. The method used a wireless accelerometer glued to the shaft, and WIFI communication system; the principle of fault detection is based on sensing the shaft instantaneous angular speed (IAS) [108]. Lucas et al. investigated mechanical behaviour during load change on the motor shaft and determine pattern change during load using a low-cost piezoelectric accelerometer. Sensors coupled to the motor’s back and front side generate a signal based on the motor vibration. For data processing, analytic RMS voltage and cross-correlation were also used to find similarities between signals; MATLAB software was used. The study notes that the load, even with the steady-state of the electrical network load, changes the vibration behaviour on its own [109,110]. The vibration sensors acquired signals containing several components, including noise signals and fault signals. Hence, signal process analysis (SPA) methods were used to analyse and classify fault signals. VST employed the SPA technique to determine and classify different faults and their severity. There are several types of SPA techniques, each able to classify different types of faults. SPA has two main principles: separation of the signal from noise and analysis of the main component, such as local projection (LP), and the use of high-order polynomials as denoising methods. The authors in [111,112,113] proposed Adaptive High-Order Local Projection (AHLP), which calculates the centroid of the neighbourhood to reduce the vibration signal noise in bearing various faults diagnoses to extract the frequency domain features (see Figure 28). Moreover, finite-element analysis (FEA) is used to obtain shaft output torque pulsation. The stochastic resonance (SR) technique enhances weak and unrecognisable transient faults in bearings, but due to the high power, SR does not detect the fault. Hence, He, Wu and Pan introduced a new method called multi-scale stochastic resonance spectrogram (MSSRS); the method focuses on the non-stationary property and deals with each time-frequency distribution. However, early bearing faults present a greater challenge for the technique.

**Figure 29:**
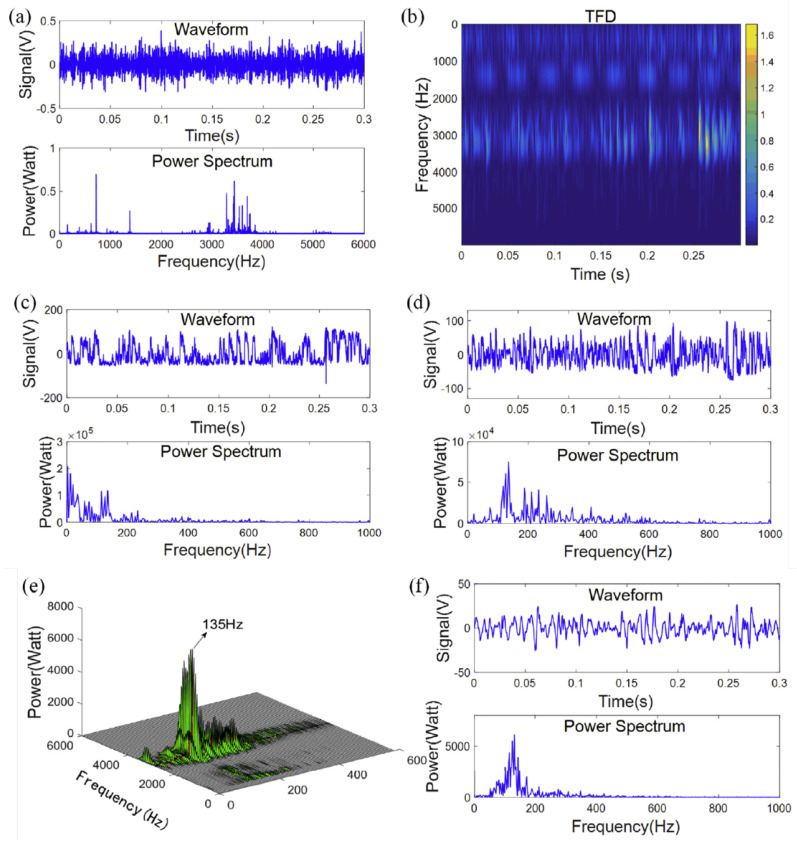
MSSRS testing result (**a**) Waveform and power spectrum, (**b**) the TFD, (**c**) the classical SR result, (**d**) the MSTSR result, (**e**) the MSSRS result, and (**f**) the optimum result in the MSSRS [113].

Early bearing fault diagnosis can be enhanced by using Statistic Resonance (SR), but it becomes difficult due to the environmental noise. Therefore, a multi-scale stochastic resonance spectrogram (MSSRS) was used. The technique is considered the non-stationary characteristic, and deals with each time-frequency distribution (TFD) (which is achieved by applying SFFT analysis) like a modulation system, instead of a time domain according to each frequency that the SR technique utilizes with each modulation, and generates a 2-D sensitive spectrogram to identify the periodic components. The method enhances the result. Figure 29a,b represent the signal and power, and because the power was high in that experiment (135.4 Hz), the stochastic resonance (SR) does not show the fault. MSSRS enhanced the result in (d), but it still has noise interference.The spectrogram is clearly visible in (e) and (f) [113]. Deep Believe Network (DBN) with quantum particle swarm optimization (QPSO) is another technique used to detect all suitable hidden layers in the vibration signal. Multiple faults is another challenge; therefore, a Stochastic Feature Selection (SFS) based on the Hidden Markov Model (HMM) is used for detection of multiple bearing faults, the method used in the calculation of the time domain, frequency domain and time-frequency domain [114,115].

**Figure 30:**
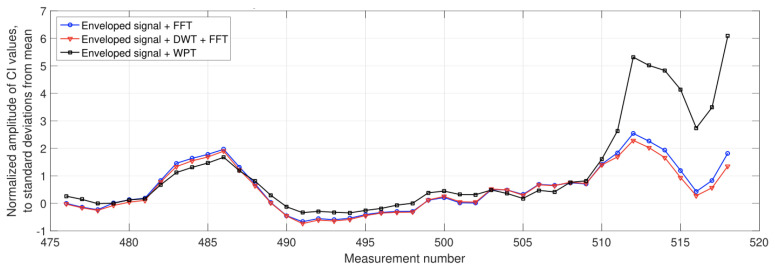
Comparative results between FFT, DWT and WPT methods [117].

This SPA review found that the time domain develops waveform generation indices, such as Peak level and RMS values, but not under specific machine loads. FFT is used to detect faults but does not identify the severity of the fault. The Artificial Neutral Network (ANN) can detect faults and classify them using a Convolution Neural Network (CNN), reducing maintenance costs. Empirical Mode Decomposition (EMD) and Hilbert transform have replaced the old envelope spectrum method, giving a better result. Moreover, the Laplace wavelet and Morley wavelet give good results using the enveloped power spectrum compared to the FFT power spectrum. Moreover, spectrum Kurtosis gave a better result in filtering noise signals. On the other hand, Wavelet Packet Transform (WPT) can filter at a higher degree and double the amplitude result compared to Fast Fourier [116,117]. Figure 30 shows the comparative result of the FFT, DWT and WPT methods.

#### 3.2.4. Wear Debris

**Figure 31:**
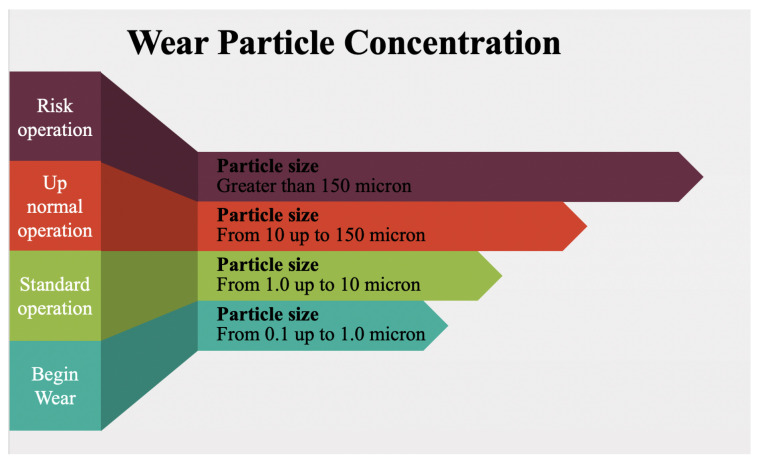
Wear debris size and credibility [122].

Wear debris is a contact-based technique used to detect and measure debris particles in the lubrication and cooling oil due to the large amount of rubbing between moving and stationary parts, ferrous and non-ferrous metal particles. The size of debris particles within the oil can be classified into three types: standard operation debris particles, sized from 1 to 10 microns, abnormal condition particles, where the size of the debris is from 10 up to 150 microns, and the size increases more than 150 microns until the machine fails (Figure 31). Different techniques are used to identify the debris particles, such as installing a magnet in the oil flow path. However, that does not detect non-ferrous metal, and the machine has to be stopped for sampling. Therefore, another technique was developed using a pulse sensor called the Inductive Coulter Counter. The sensitivity of the sensor was improved by using a two-layer planar coil and mesoscale. This method detects particles with a size of 50–75 um better than the 3D solenoid method. Another method used to enhance wear debris analysis is inductance-capacitor (LC) resonance linked to an inductive pulse debris sensor to enhance the sensitivity of detection. This method detects copper particles with resonance and non-resonance. Furthermore, ultrasonic and inductive pulse sensors can be used. The ultrasonic pulse sensor detects all solid, metallic and non-metallic debris using an acoustic focal region. Using a photodetector is another way to measure the debris particles by feeding oil into a glass tube. The photodetector detects debris particles in both non-ferrous and ferrous materials. The technique uses a LED and sound alarm [118,119,120,121]. The next section will focus on the discussion and conclusion.

## 4. Analysis of the Literature Review

The research has discussed maintenance types like breakdown, preventive and predictive approaches, and has investigated the possible definitions of the non-invasive inspection technique. These definitions are later explained by reviewing relative papers with gap identification. Maintenance aims to save lives, money, environment, production and equipment; however, breakdown maintenance does not provide this type of requirement. On the other hand, preventive maintenance would save lives, production, and equipment, but it is very costly due to the remaining useful live (RUL); therefore, predictive and condition monitoring maintenance is considered the most recommended method [110]. Condition monitoring has significantly evolved recently due to evolution in the internet of things (IoT) and cloud computing. Therefore, non-invasive becomes a significant subject for researchers because it can provide data from machines which were not equipped with vibration sensors to online condition monitoring. The non-invasive technique was introduced early and defined into two main parts. The first definition is that non-invasive inspection is when the sensing element does not need physical installation to the machine or physical installation to the machine system. The methods use laser, radio, radar, digital camera imaging, acoustic, ultrasonic waves and thermographic sensing. The second non-invasive definition is that the non-invasive inspection is when the sensing element does not have physical contact with the machine, but sensors can still be installed in the machine system. The method uses voltage, flux, current, vibration, and wear debris. However, each tool has disadvantages and advantages; therefore, tools will be explained more deeply in this section and the technique’s tools will be evaluated critically in the following paragraph.

### 4.1. Evaluation

Current, voltage and flux analysis techniques have similarity in measurement and performance. They all require physical contact with machine systems. However, those tools are suitable only for an electrically driven machine but not with machines such as fuel machine engines, and they are also not suitable for wind and steam turbines. These techniques can be influenced due to the changes in the machine power supply network and hence can produce noise in the measured signals. Moreover, these techniques are also not able to evaluate machine faults during a change in operational loads. These methods have difficulty in detecting and classifying the bearing faults [65]. It is also observed especially in flux analysis that a large number of sensors are required to get accurate results. Wear debris analysis can also be categorised as a contact-based sensing technique. Moreover, it is very accurate in finding out faults related to engine severity due to the quantity and size of detected debris particles. However, it is very difficult to find out the fault location with the help of debris analysis, especially in a complex machine system, moreover, the machine often needs to come out of operation to measure the debris particles. Similarly, the vibration technique is contact-based sensing technique. Vibration is a very well-known technique, and it uses contact based on the machine’s health detection and experimental studies due to the high accuracy of the technique in fault diagnosing, but due as a contact-sensing method, it is limited to the machines that are manufactured with equipped vibration sensors or are modified to adopt vibration sensors. Furthermore, the vibration signals transmit through the entire body of the machine, moreover, each type of vibration sensing has some drawback, such as accelerometers being sensitive to high frequency noise, and velocity sensors being sensitive to low resonant frequency and phase shift and cross noise. Furthermore, proximity sensors are impacted by electrical and electronic noise, bounded by high frequencies, and are not calibrated for unknown metal materials. Vibration is a contact-based form of sensing, however, the laser technique can apply the vibration principle to detect machine faults. This approach produces very good machine inspection results, although the laser casing reduces the risk to human, however, it is still available. The ultrasonic principle can be used both with contact and non-contact-based sensors. It is susceptible to temperature change and has resolution problems for fragile and small objects, and it gives better accuracy of measurement if the sensor and object are at the same line of sight. Due to the high measuring range, the technique is used to measure the distance of moving objects, in burglar alarms and for liquid levels, but has not been used to detect machine health. The thermographic sensing technique is very good at classifying faults due to the colour coding feature; it is also safe to use and provides fast measuring results. However, IR is significantly affected by emissivity and the reflection of the object’s surface. Furthermore, IR cannot detect through glasses, and the technique detects better over a short distance. It also works better with the low data rate process, and the thermal camera is the best, with a +/−0.02 accuracy. Similarly, the acoustic emission AE sensing technique has advantages due to the non-contact manner and the ease of installation. However, it has a drawback due to the background noise around the tested machine and the environment; moreover, the technique has difficulty separating noise from the acquired signal component. AE also cannot classify the fault location.

### 4.2. Identifying Gaps and How to Close the Gap

The work has done critical research reviewing academic publications for more than 120 published papers to identify the unaddressed challenges in machine health diagnostics to detect shaft misalignment and gearbox tooth damages via non-invasive inspection and non-contact-based methods for indoor and out-door machines. The evaluation presented that the flux, voltage and AC analyses are limited to electrical driven machines that do not suit shale oil engines and use contact-based methods. However, vibration and wear debris are contact-based techniques; they have been disregarded because the research aimed to find non-contact-based techniques. Camera imaging, thermographic, AE, and laser are non-contact measuring sensors; they have been used in machine health diagnosis before. However, there are still some limitations, such as high cost for high accuracy sensors, media separation, environmental noises, and difficulty classifying faults. A few researchers used radar sensing techniques to diagnose machine faults by simulating speakers; another study positioned the radar sensor to detect the motor’s cooling fan reflection [49,51,123]. Nevertheless, no research used radar directly and practically to detect gearbox tooth damages and shaft misalignment. Hence, the research found that the radar sensing technique has not been used previously to detect machine health diagnosis, particularly for gearbox tooth damages and shaft misalignment; therefore, a radar sensing technique is proposed to close the research gap.

### 4.3. Suitability Matrix of NII Techniques for Different Applications

The following section is a matrix table to introduce different sensing techniques by listing each technique’s key characteristics, limitations, and applications as a summary of those sensing techniques based on the research work Table 1.

## 5. Conclusions

The study aims to investigate effective techniques that can measure, monitor and diagnose machine health and machine component health during operation without interruption to machine operation and without using a space in the machine to observe the accurate result in near real-time on detecting gearbox tooth damages and shaft misalignment in a non-invasive manner. The research carried out based on a critical and comprehensive review included more than 120 published papers. The reviewed papers focused on the NII manner techniques such as flux, acoustic, ultrasonic, vibration, laser, radar, radio, imaging and thermographic techniques to discern the techniques’ strength and weaknesses with different fault categories. Additionally, the researchers classified the definition of NII into two main definitions.

The research can be concluded by way of several techniques used to diagnose machine and machine component health, however, conventional techniques have measurement limitations, such as contact-based sensing, the range of sensing objects, noise impact, cost and safety. Furthermore, the study found that no researcher has thoroughly investigated gearbox failures and shaft misalignment with the help of radar sensing to measure, monitor, and diagnose the gearbox tooth damage and shaft misalignment, during machine operation, without interruption to machine operation, and without using a space in the machine for accurate results in near real-time. Hence, this research identified a gap in the limitation of existing sensing techniques and the non-comprehensive use of radar sensing techniques in machine health monitoring and diagnosis. The excellent features of the radar sensing technique have been investigated, such as long-distance measuring range, the detection of multi-devices simultaneously, higher accuracy, and less affection of noise. Furthermore, radar sensors have tremendous capability to be used in different types of weather such as rain, dust, fog, and cloud. Radar sensing also has a non-invasive and non-contact feature. In addition, the radar signal can penetrate insulation material such as plastic and rubber, and detect objects with a high accuracy target. Moreover, radar can classify moving and stationary objects, and is costless compared to other sensing techniques. Hence, the excellent features of radar sensing illustrate that radar sensing can eliminate the research gaps. However, during the research, it was found that there is a division in defining the non-invasive technique. Therefore, the non-invasive technique has been defined as two definitions based on sensing methods, i.e., contact and non-contact sensing techniques. The radar sensing technique will use an experimental method to demonstrate the advantage of the radar sensing technique and the higher quality of sensing result. The expected result is that the radar sensing technique will enhance the machine health diagnosis process in gearbox damages and shaft misalignment. Therefore, practicians should consider the technique for future research. The contribution of the radar technique proposal was to eliminate conventional sensing technique limitations and the ignorance of using radar sensors. The proposal will add a new reliable technique to the machine health diagnosis due to the more extended measuring range and higher quality, along with the large number of features that the radar sensing technique would add to the machine health diagnosis process, and the low cost and safe use. This will inspire researchers to make the sensing technique movable and able to be attached to a drone to detect tall and remote objects such as towers and wind turbines.

## Figures and Tables

**Table 1:** Suitability matrix of NII techniques for different applications.

Technique	Key Characteristic	Limitations	Application
**Contact-Based Techniques**			
Wear debris [118,119,120,121]	Very accurate at establishing severity Low cost	Cannot locate faults Interrupts operation	Machine lubricant oil analysis
Vibration sensing [109,110,116,117]	Good response Withstand high temperatures High accuracy	Contact-based Sensitive to machine noise	Machine body and equipment measurement
Flux radiation or voltage and current [92,93,94,97,98]	Low cost Fast action Simple installation	Suitable for motors but not fuel-powered engines Impacted by a change in the supply network Weakness in tracking load change Cannot quantify severity of damage	Motor body and wiring
**Non-Contact Based Techniques**			
Ultrasonic sensing [57,58]	Insensitive to weather conditions Senses all materials Larger size provides better sensing	Sensitive to temperature changes Struggles to read small object reflection	Anti-collision Doors
Imaging camera [63,64]	non-contact No harmful radiation Operates in real-time	Heavy and large size Impacted by weather and dust High usage of data	Image monitoring Machine vibration Car speed detection
Thermographic sensing [83,84,85,86]	No- harmful radiation Life-time operation Specify fault area	Temperature interference from others surfaces	Human body detection Machine and equipment heat
Radar sensing [46,48,49,50,51,52]	Insensitive to weather conditions Multiple objects at a time Easy installation Fast data acquisition Longer measuring range Detect different types of materials	High cost No fault classification Signal spreads Hard to classify close objects	Detect objects and motions Object speed and size Car’s sensors Autonomous car Door
Acoustic emission [67,72,73]	Non-contact High sensitivity Low cost Real-time monitoring	Noise impact No fault classification	Music ditemize Machine noise sensing
Laser sensing [89,90,91]	Non-contact Multiple object detection Long measuring range	No fault classification Cost Eye risk Affected by the weather	Machine vibration sensing

## Data Availability

Not applicable.
